# Supplementary material for: Structural Polymorphism of Guanine Quadruplex-Containing Regions in Human Promoters
Source: Int J Mol Sci. 2022 Dec 16;23(24):16020. doi: 10.3390/ijms232416020 (PMC9786302; doi:10.3390/ijms232416020)
Supplement: Supplementary file 1 [file ijms-23-16020-s001.zip › ijms-2048445-supplementary.pdf]

Supplementary Information

Structural polymorphism of guanine quadruplex-containing regions in human promoters

Christopher Hennecker, Lynn Yamout, Chuyang Zhang, Chenzhi Zhao, David Hiraki, Nicolas Moitessier, and Anthony Mittermaier

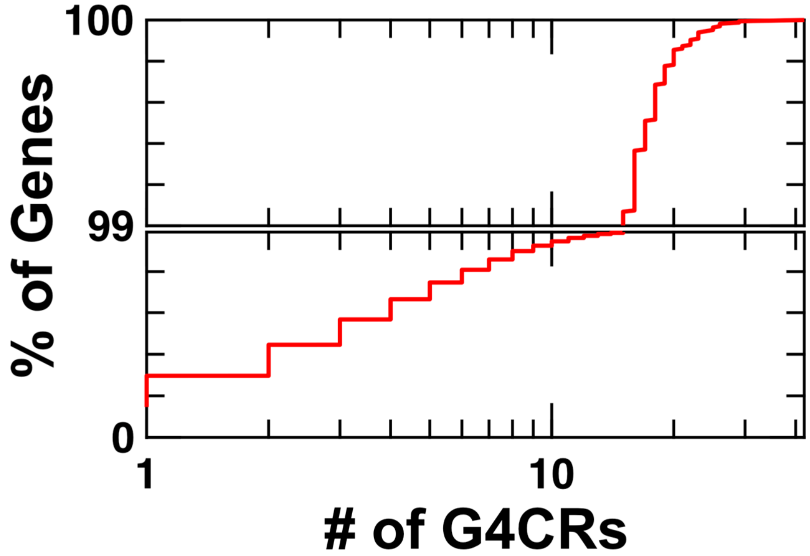

Figure S1: Cumulative plot of the number of G4CRs for human genes considering both coding and non-coding strands. The number of G4CRs is plotted on a logarithmic scale. The bottom panel represents the first 99% of genes whereas the top panel represents the top 1% of genes.

Figure S2a. Animals

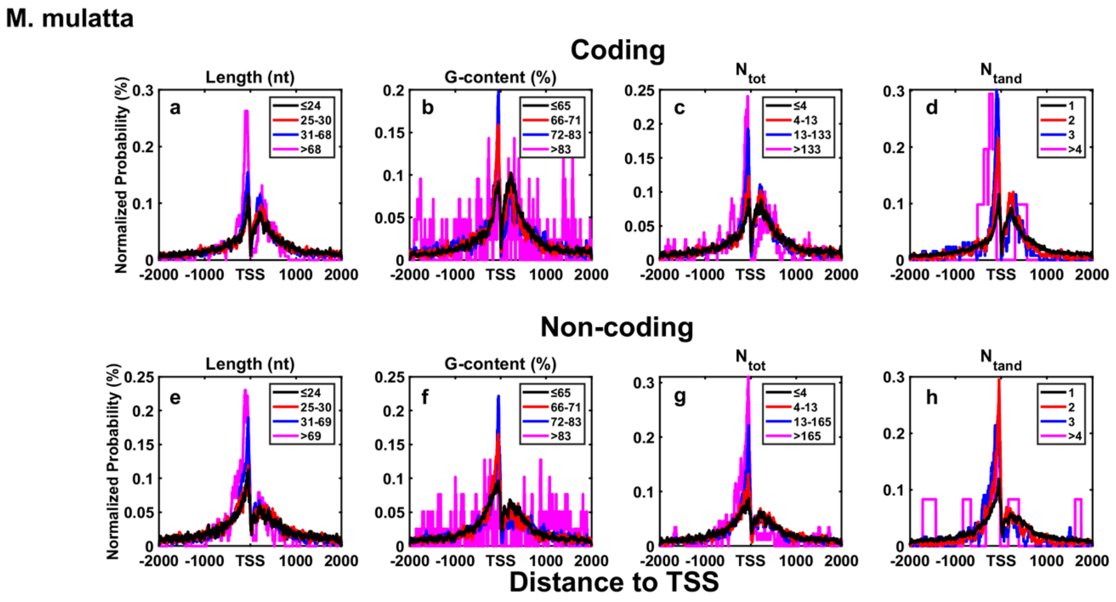

M. musculus

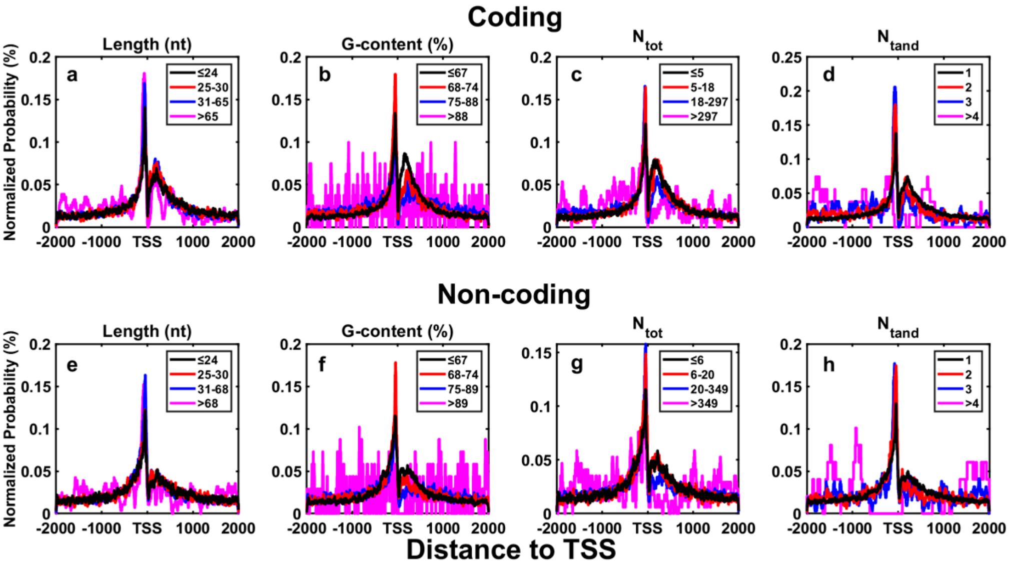

R. norvegicus

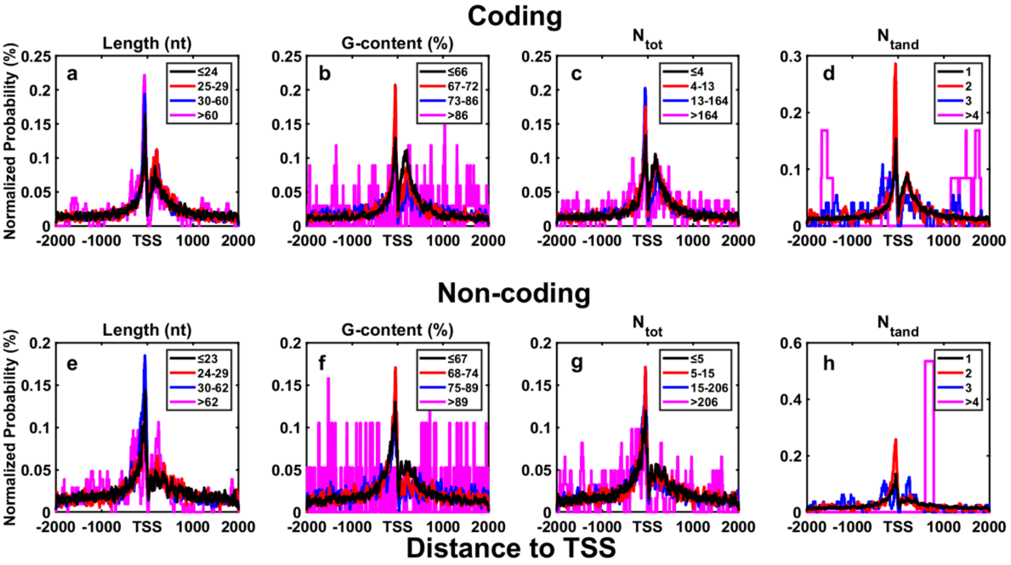

C. familiaris

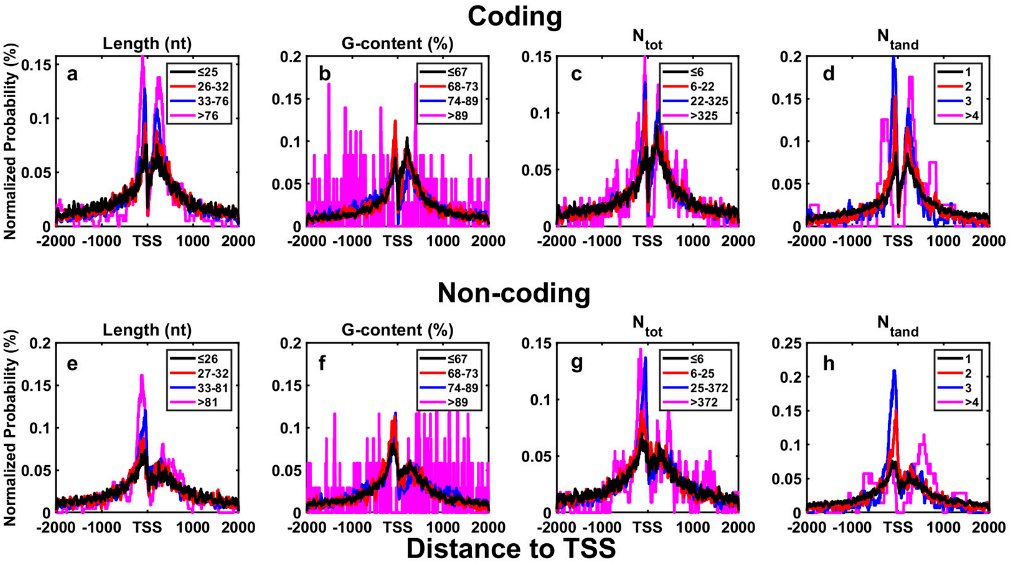

G. gallus

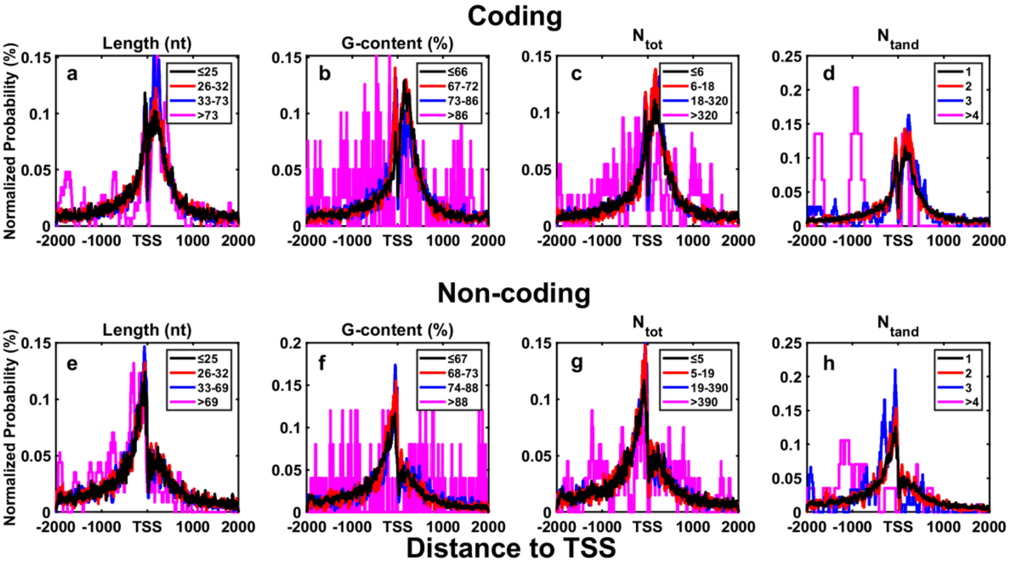

Figure S2b. Invertebrates

D. melanogaster

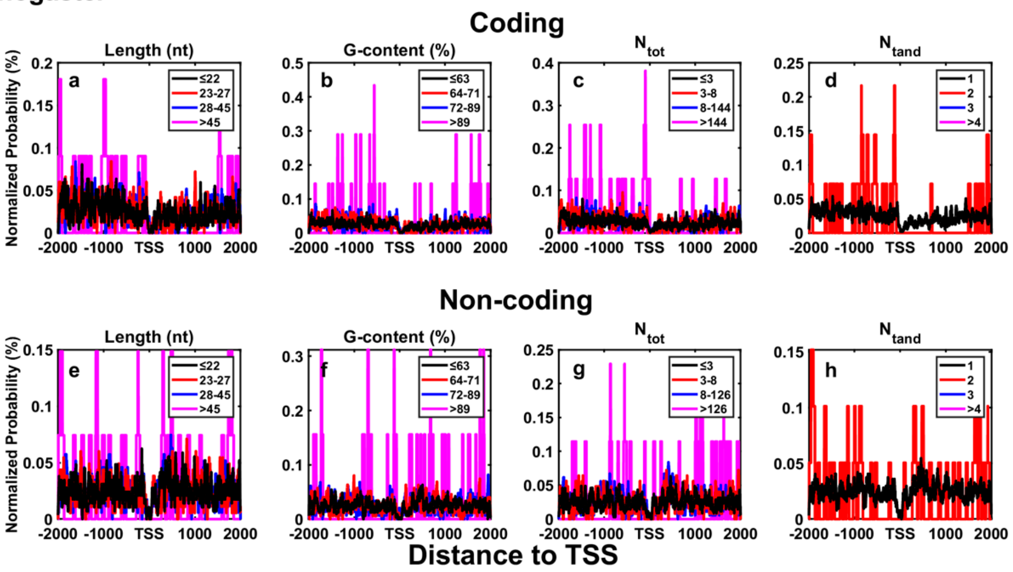

A. mellifera

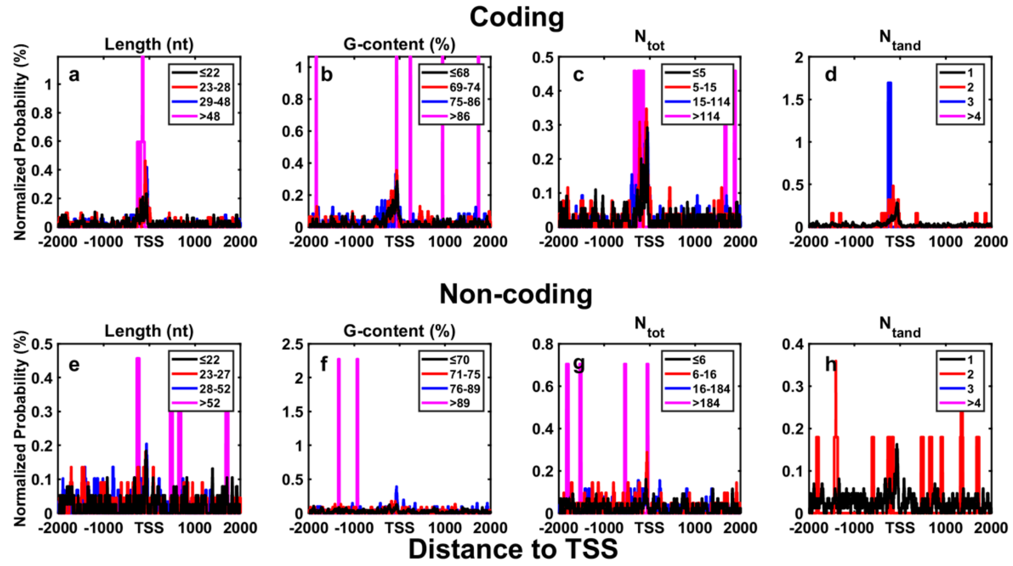

D. rerio

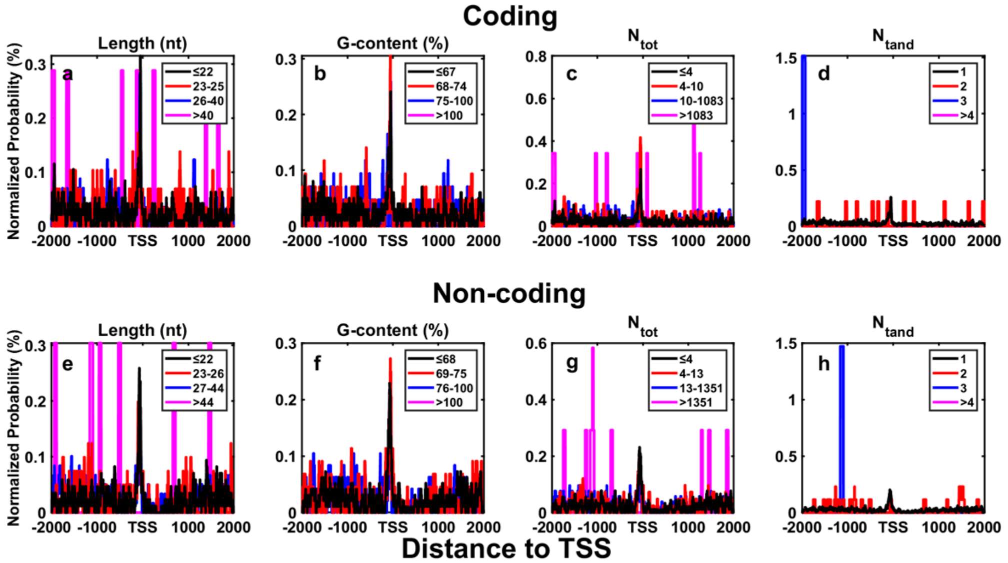

C. elegans

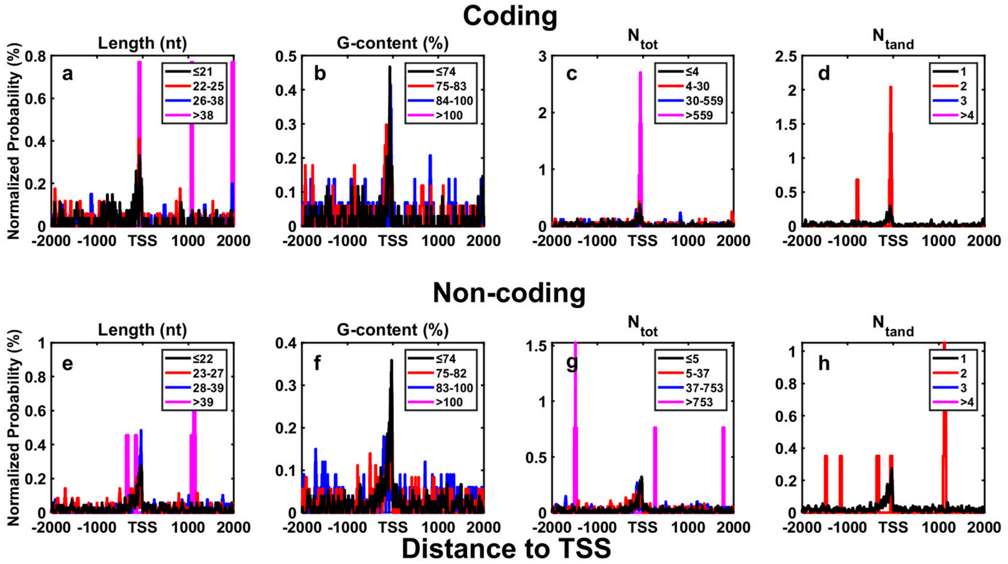

Figure S2c. Plants

A. thaliana

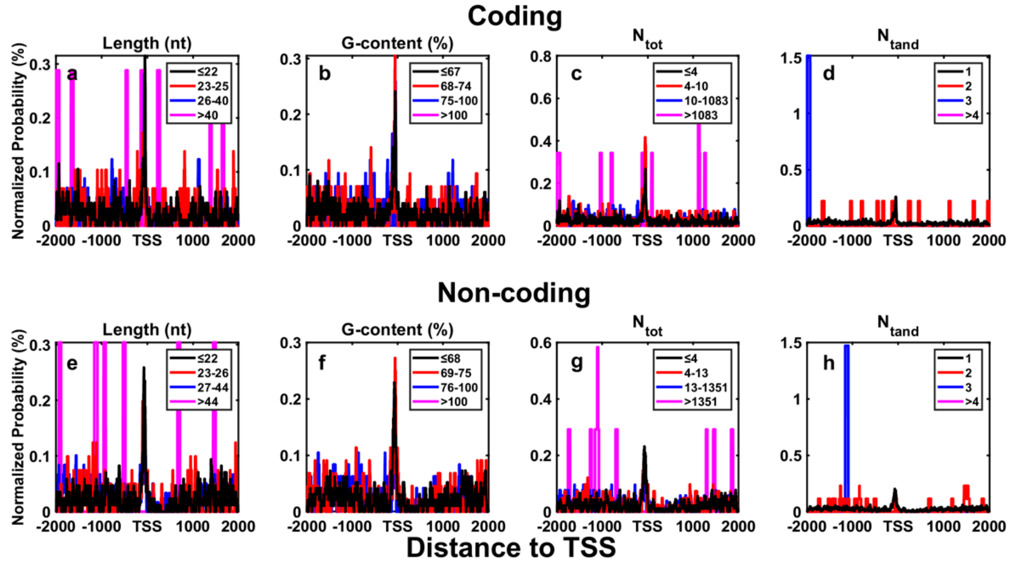

Z. mays

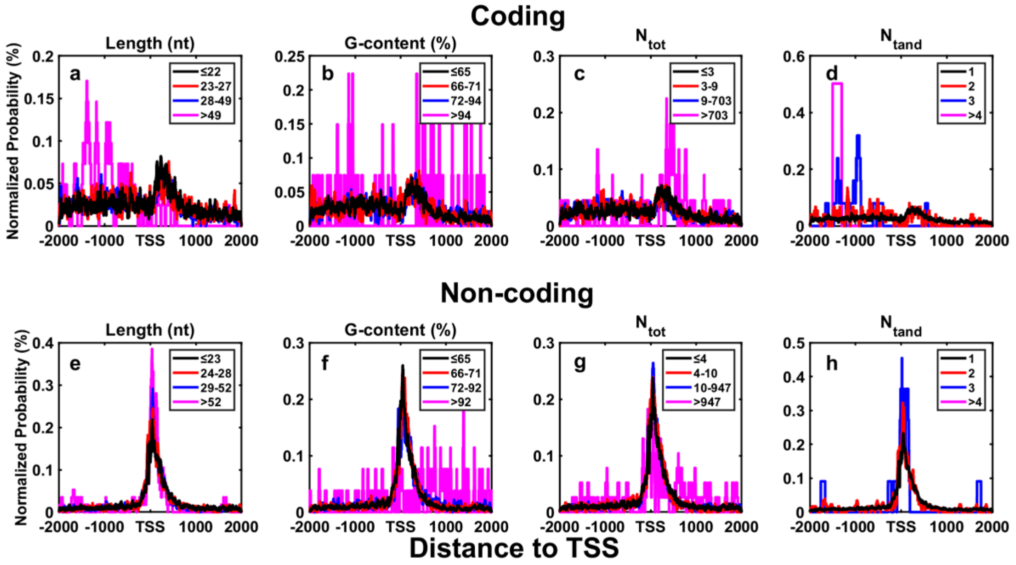

Figure S2d. Fungi

S. cerevisiae

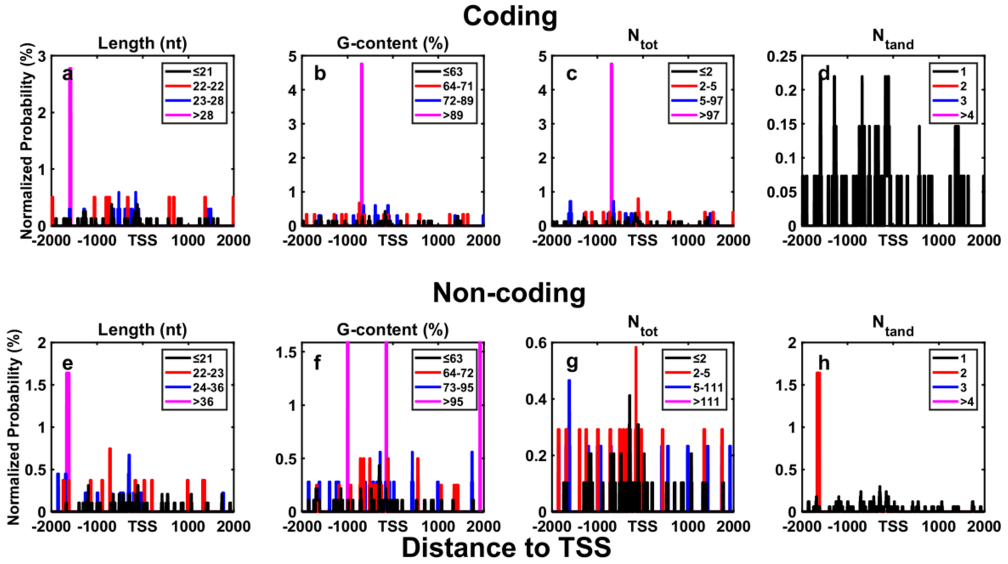

S. pombe

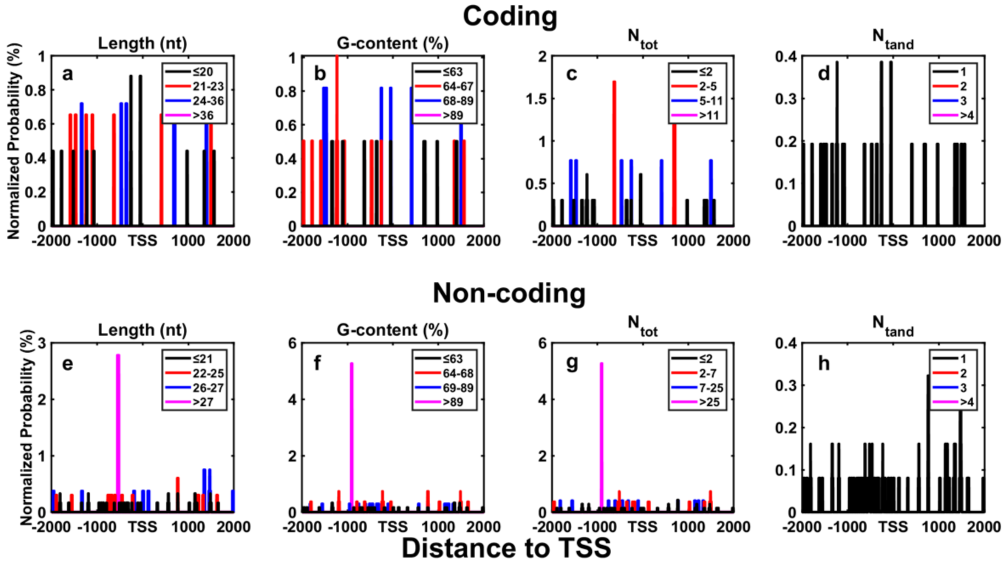

Figure S2e. Protozoa

P. falciparum

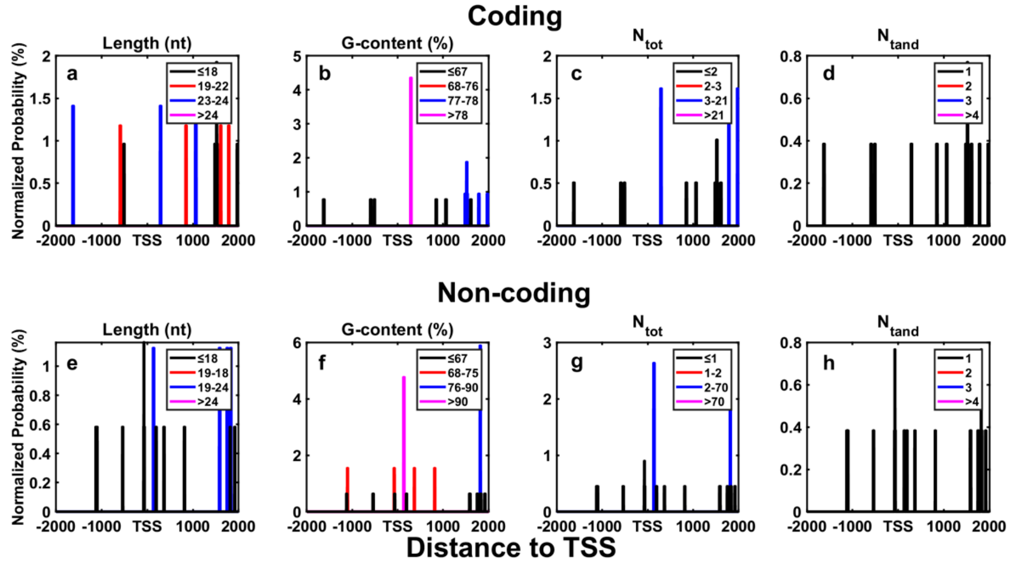

Figure S2f. Shuffled

Shuffled

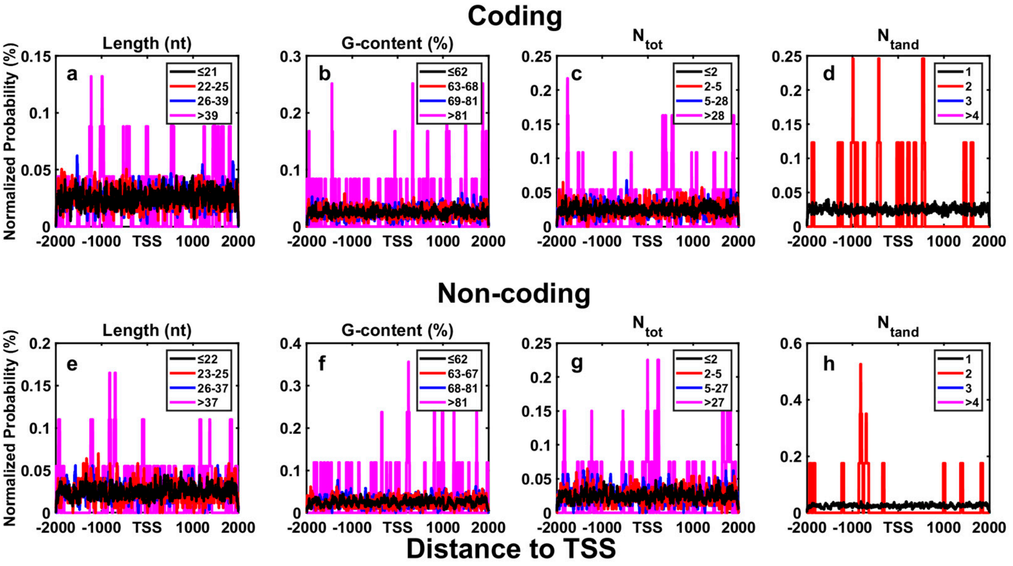

Figure S2: Likelihood that a residue lies within a G4CR possessing certain characteristics, plotted as a function of the residue's position relative to the transcription start site (TSS), for ***a)*** *animals,* ***b)*** *invertebrates,* ***c)*** *plants,* ***d)*** *fungi,* ***e)*** *protozoa,* ***f)*** *shuffled, promoter sequences. Colours represent the bottom 50% of G4CRs (black), 50-75% of G4CR, 76-99%, and top 1% of G4CRs. Specific values are given in the legend of each panel. Panels a-d are for the coding strand (Length, G-content,* N_{tot}*, and* N_{tand} *respectively). Panels e-h are for the non-coding strand (Length, G-content,* N_{tot}*, and* N_{tand} *respectively).*

Table S1: Statistics on analysis of the coding strand of eukaryote promoters. Length, %guanosine, and N_{tot} are reported as their median values.

**Coding Strand**						
**Species**	**# Promoters**	**# G4CRs**	**#G4 motifs**	**Length**	**%guanosine**	**N_{tot}**
H. sapiens	29598	61012	921566	25	65	4
M. mulatta	9575	17619	241709	24	65	4
M. musculus	25111	35503	857790	24	67	5
R. norvegicus	12601	15768	256013	24	66	4
C. familiaris	7545	20590	572821	25	67	6
G. gallus	6127	15340	375948	25	66	6
D. melanogaster	16972	2339	25600	22	63	3
A. mellifera	6493	464	6223	22	68	5
D. rerio	10728	800	37917	22	67	4
C. elegans	7120	290	16698	22	74	4
A. thaliana	10728	800	37917	22	67	4
Z. mays	17081	6165	188183	22	65	3
S. cerevisiae	5117	63	490	21	63	2
S. pombe	4802	24	79	21	64	2
P. falciparum	5597	13	46	20	76	2

Table S2: Statistics on analysis of the non-coding strand of eukaryote promoters. Length, %guanosine, and N_{tot} are reported as their median values.

**Non-coding Strand**						
**Species**	**# Promoters**	**# G4CRs**	**#G4 motifs**	**Length**	**%guanosine**	**N_{tot}**
H. sapiens	29598	50524	833421	24	65	4
M. mulatta	9575	14818	211580	24	65	4
M. musculus	25111	30783	862230	24	67	6
R. norvegicus	12601	13324	238733	23	67	5
C. familiaris	7545	18475	645158	26	67	6
G. gallus	6127	11463	322532	25	67	5
D. melanogaster	16972	2726	26668	22	63	3
A. mellifera	6493	373	6851	22	70	6
D. rerio	10728	790	49703	22	68	4
C. elegans	7120	616	33230	22	74	5
A. thaliana	10728	790	49703	22	68	4
Z. mays	17081	10195	360194	23	65	4
S. cerevisiae	5117	79	584	21	64	2
S. pombe	4802	56	312	21	63	2
P. falciparum	5597	14	84	18	67	1

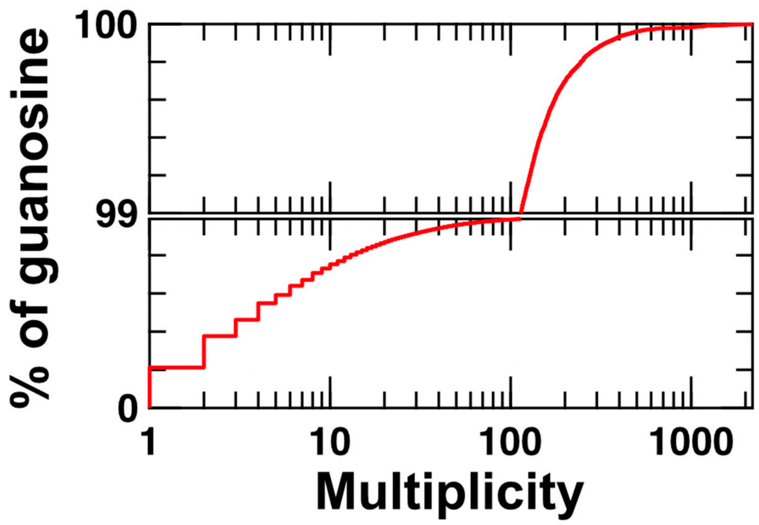

Figure S3: Cumulative plot of multiplicity for guanines participating in at least one G4 structure in a G4CR. Multiplicity is plotted on a logarithmic scale. The bottom panel represents the first 99% of multiplicities whereas the top panel represents the top 1% of multiplicities.

Table S3: Statistics on all the G4CRs found in the human MYC promoter discussed in the main text and Figure 7. G4CRs found on the coding strand are indicated by "c" and G4CRs found on the non-coding strand are indicated by "n".

MYC								
**G4CR**	**Length (nt)**	**G-Content (%)**	**N_{tot}**	**N_{tand}**	**Distance to TSS**	**T_m (min)**	**T_m (median)**	**T_m (max)**
1c	27	74	7	1	-887	50.9	54.5	60.7
2c	54	69	21	1	1654	50.6	53.0	60.7
1n	25	64	6	1	-1034	56.3	61.2	64.5
2n	59	64	85	2	-94	50.7	60.3	84.1
3n	31	58	4	1	321	50.7	61.4	69.2
4n	21	62	1	1	1309	50.7	50.7	50.7

Table S4: Statistics on all the G4CRs found in the human VEGFA promoter discussed in the main text and Figure 7. G4CRs found on the coding strand are indicated by "c" and G4CRs found on the non-coding strand are indicated by "n".

**VEGFA**								
**G4CR**	**Length (nt)**	**G-Content (%)**	**N_{tot}**	**N_{tand}**	**Distance to TSS**	**T_m (min)**	**T_m (median)**	**T_m (max)**
1c	23	74	20	1	-1786	50.7	57.4	80.7
2c	26	65	2	1	-1322	54.5	56.6	58.7
3c	30	70	19	1	-59	52.2	66.8	78.4
4c	19	79	4	1	634	55.0	59.5	64.1
5c	28	71	11	1	1575	50.6	53.0	58.7
1n	31	71	9	1	-1085	52.2	55.0	64.1
2n	19	74	5	1	-388	55.0	58.4	72.6
3n	22	64	2	1	1328	50.7	59.3	67.9
4n	22	64	1	1	1675	53.7	537	53.7
5n	20	70	3	1	1703	51.6	52..6	55.0
6n	27	67	6	1	1739	55.0	74.1	80.7
7n	19	79	6	1	1866	52.6	56.7	60.7

Table S5: Statistics on all the G4CRs found in the human BCL2 promoter discussed in the main text and Figure 7. G4CRs found on the coding strand are indicated by "c" and G4CRs found on the non-coding strand are indicated by "n".

**BCL2**								
**G4CR**	**Length (nt)**	**G-Content (%)**	**N_{tot}**	**N_{tand}**	**Distance to TSS**	**T_m (min)**	**T_m (median)**	**T_m (max)**
1c	21	67	1	1	-329	55.0	55.0	55.0
2c	33	70	16	1	-198	50.6	54.6	58.4
3c	25	72	12	1	1359	55.8	60.6	64.9
1n	21	76	6	1	-544	50.7	55.3	56.5
2n	20	70	3	1	-292	51.6	52.6	55.0
3n	80	69	40	3	-11	50.7	57.5	69.9

Table S6: Statistics on all the G4CRs found in the human KIT promoter discussed in the main text and Figure 7. G4CRs found on the coding strand are indicated by "c" and there are no G4CRs found on the non-coding strand of the KIT promoter.

KIT								
**G4CR**	**Length (nt)**	**G-Content (%)**	**N_{tot}**	**N_{tand}**	**Distance to TSS**	**T_m (min)**	**T_m (median)**	**T_m (max)**
1c	33	73	14	1	-90	50.7	52.6	76.5
2c	22	73	6	1	-51	50.7	56.7	66.8
3c	31	65	9	1	1220	52.2	55.6	64.5

Table S7: Statistics on all the G4CRs found in the human KRAS promoter discussed in the main text and Figure 7. G4CRs found on the coding strand are indicated by "c" and G4CRs found on the non-coding strand are indicated by "n".

KRAS								
**G4CR**	**Length (nt)**	**G-Content (%)**	**N_{tot}**	**N_{tand}**	**Distance to TSS**	**T_m (min)**	**T_m (median)**	**T_m (max)**
1c	62	69	103	2	343	50.6	56.3	75.0
1n	52	65	25	2	-165	50.7	56.3	64.9
2n	26	58	2	1	-120	51.9	55.1	58.4
3n	31	71	3	1	225	53.0	63.4	66.8
4n	41	69	48	1	595	50.7	55.8	65.8

Webserver Description

The webserver has top navigation bar with five buttons: **Input Seq**, **Seq Overview**, **G4 containing region**, **help**, and **contact us** (Figure S4 below).

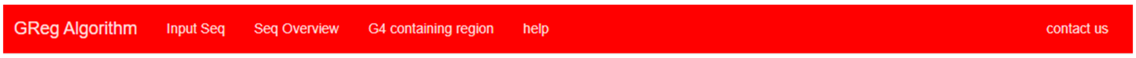

Figure S4. *The top navigation bar of the webserver.*

When a user first accesses the site, they are automatically directed to the **Input Seq** page, which contains a textbox allowing a DNA sequence to be entered by typing or copying and pasting. In addition, the user is required to enter the parameters to be used in the calculation: the maximum loop length, the maximum bulge size, and the minimum estimated melting temperature (°C) for a G4 to be counted among the final ensemble. As well, the user is given the option of calculating G4regions for the complement of the entered sequence and must give the sequence an identifying label (the example in Figure S5 below is taken from Figure 3 in the text).

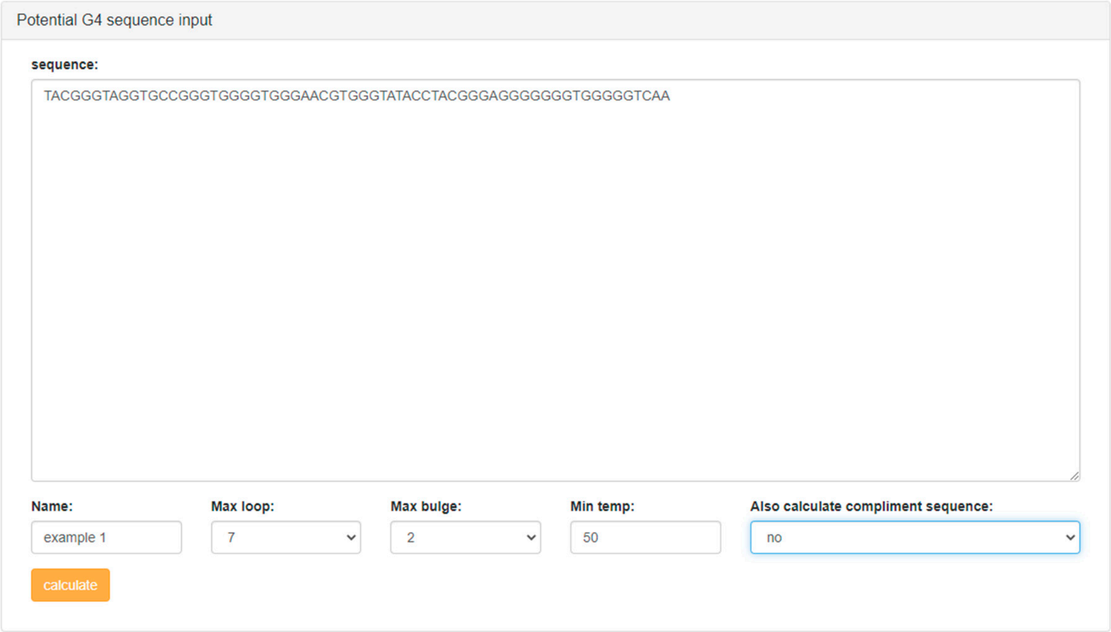

Figure S5. *The* ***Input Seq*** *page.*

Clicking the calculate button at the bottom of the screen initiates the calculate and brings the user to the **Seq Overview** page. The top part of this page contains an interactive Multiplicity Chart that plots folding multiplicity (the number of structural distinct G4s that incorporate a particular G residue into the core) as a function of residue number. Values are plotted in the same order as the sequence entered (i.e. 5' to 3'). Note that if the calculate complement option is set to yes, the values for the complementary strand will be plotted 3' to 5', so that adjacent positions in the DNA duplex will be plotted adjacently in the chart.

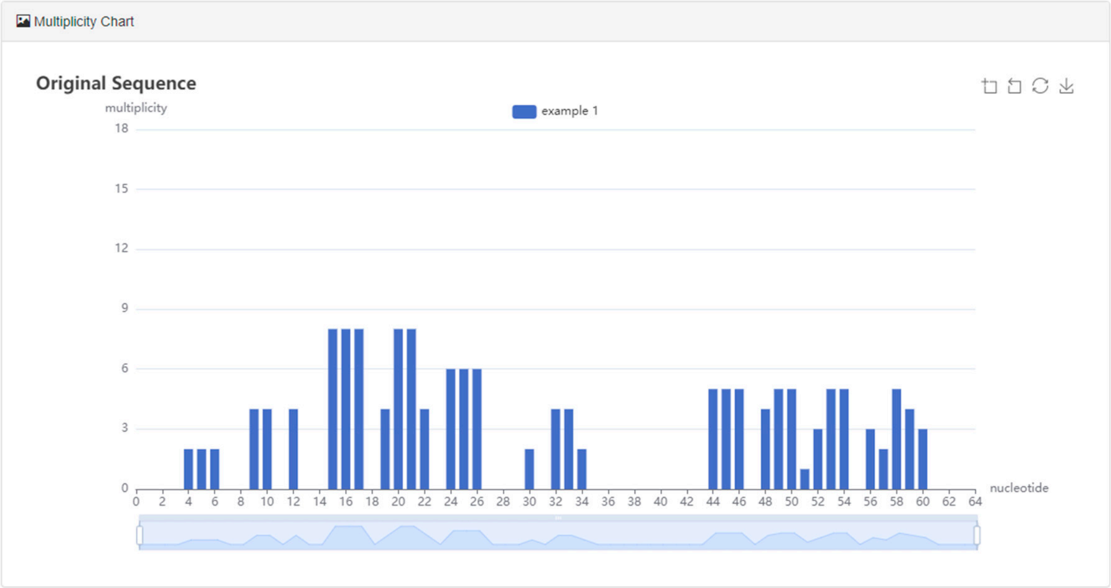

Figure S6. *The **Seq Overview** page (top)*

The bottom of the figure contains a slider that allows the user to zoom in on specific regions of the nucleotide sequence. Additional sequences can be added by clicking the **Input Seq** button in the navigation bar (for example here, the Pu27 sequence from the human *MYC* promoter). All sequences can be plotted simultaneously in the Multiplicity Chart and can be toggled between visible/hidden by clicking the corresponding button at the top of the plot.

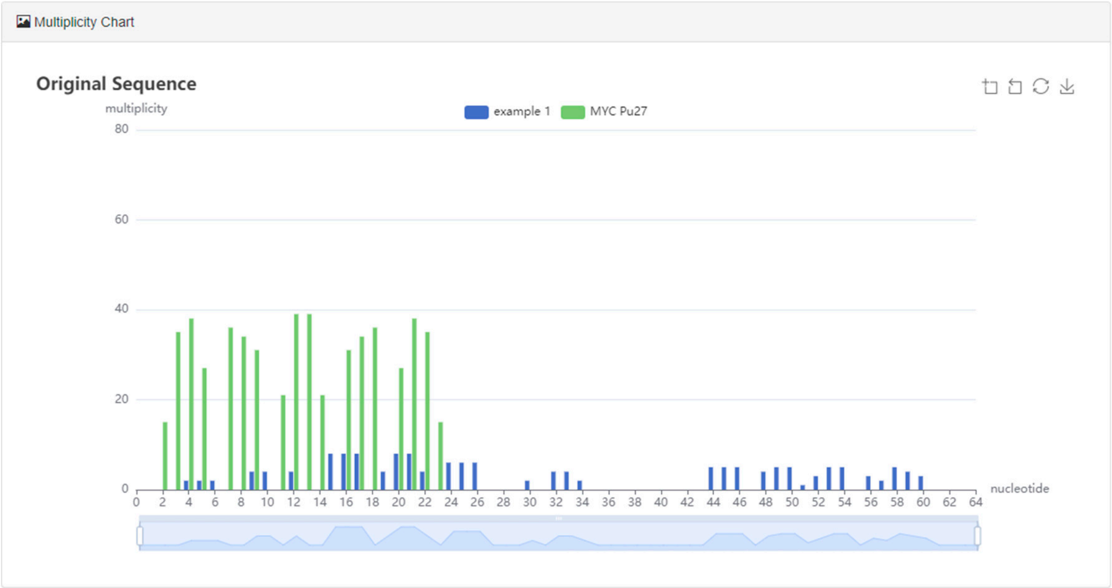

Figure S7. *The **Seq Overview** page (top), with multiple sequences.*

The bottom of the **Seq Overview** page contains the list of sequences entered by the user. The right of each sequence are **details** and **delete** buttons.

Prediction G-Quadruplex Result Overview

ID	name	sequence	number of G4CR	operation
1	example 1	TACGGGTAGGTGCCGGGTGGGGTGGGAACGTGGGTATACCTACGGGAGGGGGGGTGGGGGTCAA	2	details delete
2	MYC Pu27	TGGGGAGGGTGGGGAGGGTGGGGAAGG	1	details delete

Figure S8. *The* ***Seq Overview*** *page (bottom)*

The **delete** button deletes the particular sequence. The **details** button lists all G4CRs within the sequence, the multiplicity values of each position with the G4CR, the location of the G4CR within the sequence, the length of the G4CR, the guanine content of the G4CR, the total number of different G4s than can be formed within the G4CR, the total number that can form simultaneously (in tandem), and the minimum, median, and maximum estimated T_m values of the G4s within each G4CR. Example 1 contains two G4CRs:

Each G4 containing region

ID	name	sequence	multiplicity	position	length	%G	# of quadruplexes	# of tandem	Tm^{est}_{min}	Tm^{est}_{median}	Tm^{est}_{max}	operation
1	example 1	GGGTAGGTGCCGGGTGGGGTGGGAACGTGGG	[2, 2, 2, 0, 0, 4, 4, 0, 4, 0, 0, 8, 8, 8, 0, 4, 8, 8, 4, 0, 6, 6, 6, 0, 0, 0, 2, 0, 4, 4, 2]	4 to 34	31	65	8	1	52.6	58.3	70.7	details
2	example 1	GGGAGGGGGGGTGGGGG	[5, 5, 5, 0, 4, 5, 5, 1, 3, 5, 5, 0, 3, 2, 5, 4, 3]	44 to 60	17	88	5	1	64.1	80.7	89.9	details

Figure S9. *Sequence* ***details*** *output.*

The **G4 containing region** button in the navigation bar provides a similar table that includes all of the user's sequences. The **details** button associated with each G4CR gives a list of every G4 formed by a G4CR, with core guanine residues indicated with capital letters, together with the starting and ending position and estimated T_m. Shown below are the details for the first G4CR of example 1:

Individual G-Quadruplex

Number	sequence	start position	end position	estimated Tm
1	GGGtaGGtGccGGGtGGGgtgggaacgtggg	4	21	58.3
2	gggtaGGtGccGGGtGGGgtGGGaacgtggg	9	26	58.3
3	gggtaGGtGccGGGtgGGGtGGGaacgtggg	9	26	58.3
4	GGGtaGGtGccGGGtgGGGtgggaacgtggg	4	22	52.6
5	gggtaggtgccGGGtGGGgtGGGaacGtGGg	15	33	55.0
6	gggtaggtgccGGGtgGGGtGGGaacGtGGg	15	33	55.0
7	gggtaggtgccGGGtGGGgtGGGaacgtGGG	15	34	70.7
8	gggtaggtgccGGGtgGGGtGGGaacgtGGG	15	34	70.7

Figure S10. *G4CR* ***details*** *output.*

The **Download Result** button located at the top of the **Seq Overview** page opens a new tab with the entirety of the results listed as text. The example 1 and MYC Pu27 datasets give:

```
>>> example 1 | number of G-quadruplex containing regions: 2
0 0 0 2 2 2 0 0 4 4 0 4 0 0 8 8 8 0 4 8 8 4 0 6 6 6 0 0 0 2 0 4 4 2 0 0 0 0 0 0 0 0 0 5 5 5 0 4 5 5 1 3 5 5 0 3 2 5 4 3 0 0 0 0
>> example 1_G4CR_1 | position: 4-34 | number of nucleotides: 31 | percentage G content: 65 | total number of quadruplex(es)
could form 8 | number of tandem repeat(s) 1 | maximum estimated melting temperature: 70.7 | median estimated melting
temperature: 58.3 | minimum estimated melting temperature:52.6
GGGTAGGTGCCGGGT GGGGTGGGAACGTGG G
[2, 2, 2, 0, 0, 4, 4, 0, 4, 0, 0, 8, 8, 8, 0, 4, 8, 8, 4, 0, 6, 6, 6, 0, 0, 0, 2, 0, 4, 4, 2]
GGGtaGGtGccGGGtGGGgtgggaacgtggg | start position:4 | end position:21 | estimated melting temperature:58.3
gggtaGGtGccGGGtGGGgtGGGaacgtggg | start position:9 | end position:26 | estimated melting temperature:58.3
gggtaGGtGccGGGtgGGGtGGGaacgtggg | start position:9 | end position:26 | estimated melting temperature:58.3
GGGtaGGtGccGGGtgGGGtgggaacgtggg | start position:4 | end position:22 | estimated melting temperature:52.6
gggtaggtgccGGGtGGGgtGGGaacGtGGg | start position:15 | end position:33 | estimated melting temperature:55.0
gggtaggtgccGGGtgGGGtGGGaacGtGGg | start position:15 | end position:33 | estimated melting temperature:55.0
gggtaggtgccGGGtGGGgtGGGaacgtGGG | start position:15 | end position:34 | estimated melting temperature:70.7
gggtaggtgccGGGtgGGGtGGGaacgtGGG | start position:15 | end position:34 | estimated melting temperature:70.7

>> example 1_G4CR_2 | position: 44-60 | number of nucleotides: 17 | percentage G content: 88 | total number of quadruplex(es)
could form 5 | number of tandem repeat(s) 1 | maximum estimated melting temperature: 89.9 | median estimated melting
temperature: 80.7 | minimum estimated melting temperature:64.1
GGGAGGGGGGGTGGG GG
[5, 5, 5, 0, 4, 5, 5, 1, 3, 5, 5, 0, 3, 2, 5, 4, 3]
GGGaGGGgGGGtGGGgg | start position:44 | end position:58 | estimated melting temperature:89.9
GGGaGGGgGGGtgGGGg | start position:44 | end position:59 | estimated melting temperature:84.1
GGGaGGGgGGGtggGGG | start position:44 | end position:60 | estimated melting temperature:80.7
GGGaGGGggGGtGgGGG | start position:44 | end position:60 | estimated melting temperature:64.1
GGGagGGGgGGtGgGGG | start position:44 | end position:60 | estimated melting temperature:64.1
```

continued below

>>> MYC Pu27 | number of G-quadruplex containing regions: 1
0 15 35 38 27 0 36 34 31 0 21 39 39 21 0 31 34 36 0 27 38 35 15 0 0 0 0
>> MYC Pu27_G4CR_1 | position: 2-23 | number of nucleotides: 22 | percentage G content: 82 | total number of quadruplex(es) could form 46 | number of tandem repeat(s) 1 | maximum estimated melting temperature: 84.1 | median estimated melting temperature: 66.4 | minimum estimated melting temperature:51.6
GGGGAGGGTGGGGAG GGTGGGG
[15, 35, 38, 27, 0, 36, 34, 31, 0, 21, 39, 39, 21, 0, 31, 34, 36, 0, 27, 38, 35, 15]
gGGGaGGGtGGGgaGGGtgggg | start position:3 | end position:18 | estimated melting temperature:84.1
gGGGaGGGtgGGGaGGGtgggg | start position:3 | end position:18 | estimated melting temperature:84.1
ggggaGGGtGGGgaGGGtGGGg | start position:7 | end position:22 | estimated melting temperature:84.1
ggggaGGGtgGGGaGGGtGGGg | start position:7 | end position:22 | estimated melting temperature:84.1
GGGgaGGGtGGGgaGGGtgggg | start position:2 | end position:18 | estimated melting temperature:78.3
GGGgaGGGtgGGGaGGGtgggg | start position:2 | end position:18 | estimated melting temperature:78.3
ggggaGGGtGGGgaGGGtgGGG | start position:7 | end position:23 | estimated melting temperature:78.3
ggggaGGGtgGGGaGGGtgGGG | start position:7 | end position:23 | estimated melting temperature:78.3
gGGGaGGGtGGGgagGGtGggg | start position:3 | end position:20 | estimated melting temperature:60.7
gGGGaGGGtgGGGagGGtGggg | start position:3 | end position:20 | estimated melting temperature:58.3
gggGaGGgtgGGGaGGGtGGGg | start position:5 | end position:22 | estimated melting temperature:60.7
gggGaGGgtGGGgaGGGtGGGg | start position:5 | end position:22 | estimated melting temperature:58.3
GGGgaGGGtGGGgagGGtGggg | start position:2 | end position:20 | estimated melting temperature:55.0
GGGgaGGGtgGGGagGGtGggg | start position:2 | end position:20 | estimated melting temperature:52.6
gGGGaGGGtGGGgaggGtGGgg | start position:3 | end position:21 | estimated melting temperature:58.3
gGGGaGGGtgGGGaggGtGGgg | start position:3 | end position:21 | estimated melting temperature:55.0
ggGGaGggtgGGGaGGGtGGGg | start position:4 | end position:22 | estimated melting temperature:58.3
ggGGaGggtGGGgaGGGtGGGg | start position:4 | end position:22 | estimated melting temperature:55.0
gggGaGGgtgGGGaGGGtgGGG | start position:5 | end position:23 | estimated melting temperature:55.0
gggGaGGgtGGGgaGGGtgGGG | start position:5 | end position:23 | estimated melting temperature:52.6
GGGgaGGGtGGGgaggGtGGgg | start position:2 | end position:21 | estimated melting temperature:52.6
gGGGaGGGtGGGgagggtGGGg | start position:3 | end position:22 | estimated melting temperature:75.0
gGGGaGGGtggggaGGGtGGGg | start position:3 | end position:22 | estimated melting temperature:75.0
gGGGagggtgGGGaGGGtGGGg | start position:3 | end position:22 | estimated melting temperature:75.0
gGGGaGGGtgGGGagggtGGGg | start position:3 | end position:22 | estimated melting temperature:70.7
gGGGagggtGGGgaGGGtGGGg | start position:3 | end position:22 | estimated melting temperature:70.7
gGGGaGGGtgggGaGGgtGGGg | start position:3 | end position:22 | estimated melting temperature:52.6
gGGGagGGtGgggaGGGtGGGg | start position:3 | end position:22 | estimated melting temperature:52.6
gGGGaGGGtggGGaGggtGGGg | start position:3 | end position:22 | estimated melting temperature:51.6
gGGGaggGtGGggaGGGtGGGg | start position:3 | end position:22 | estimated melting temperature:51.6
ggGGaGggtgGGGaGGGtgGGG | start position:4 | end position:23 | estimated melting temperature:52.6
GGGgagggtgGGGaGGGtGGGg | start position:2 | end position:22 | estimated melting temperature:73.7
GGGgaGGGtGGGgagggtGGGg | start position:2 | end position:22 | estimated melting temperature:69.2
GGGgaGGGtggggaGGGtGGGg | start position:2 | end position:22 | estimated melting temperature:69.2
GGGgagggtGGGgaGGGtGGGg | start position:2 | end position:22 | estimated melting temperature:69.2
GGGgaGGGtgGGGagggtGGGg | start position:2 | end position:22 | estimated melting temperature:64.9
gGGGaGGGtGGGgagggtgGGG | start position:3 | end position:23 | estimated melting temperature:73.7
gGGGaGGGtgGGGagggtgGGG | start position:3 | end position:23 | estimated melting temperature:69.2
gGGGaGGGtggggaGGGtgGGG | start position:3 | end position:23 | estimated melting temperature:69.2
gGGGagggtgGGGaGGGtgGGG | start position:3 | end position:23 | estimated melting temperature:69.2
gGGGagggtGGGgaGGGtgGGG | start position:3 | end position:23 | estimated melting temperature:64.9
GGGgaGGGtGGGgagggtgGGG | start position:2 | end position:23 | estimated melting temperature:67.9
GGGgagggtgGGGaGGGtgGGG | start position:2 | end position:23 | estimated melting temperature:67.9
GGGgaGGGtgGGGagggtgGGG | start position:2 | end position:23 | estimated melting temperature:63.4
GGGgaGGGtggggaGGGtgGGG | start position:2 | end position:23 | estimated melting temperature:63.4
GGGgagggtGGGgaGGGtgGGG | start position:2 | end position:23 | estimated melting temperature:63.4

Figure S11. *Download Result output.*

The **Delete Data** button on the **Seq Overview** page deletes all sequences. The **help** button on the navigation bar reproduces this description of the webserver. The **contact us** button has the email address of the corresponding author and a link to the lab webpage. For security reasons, each of the users has a session of 14 days to access their input and calculated data once they first visit our server. The data is kept in our database for 14 days until the session expired, and all the information are automatically and permanently deleted.
